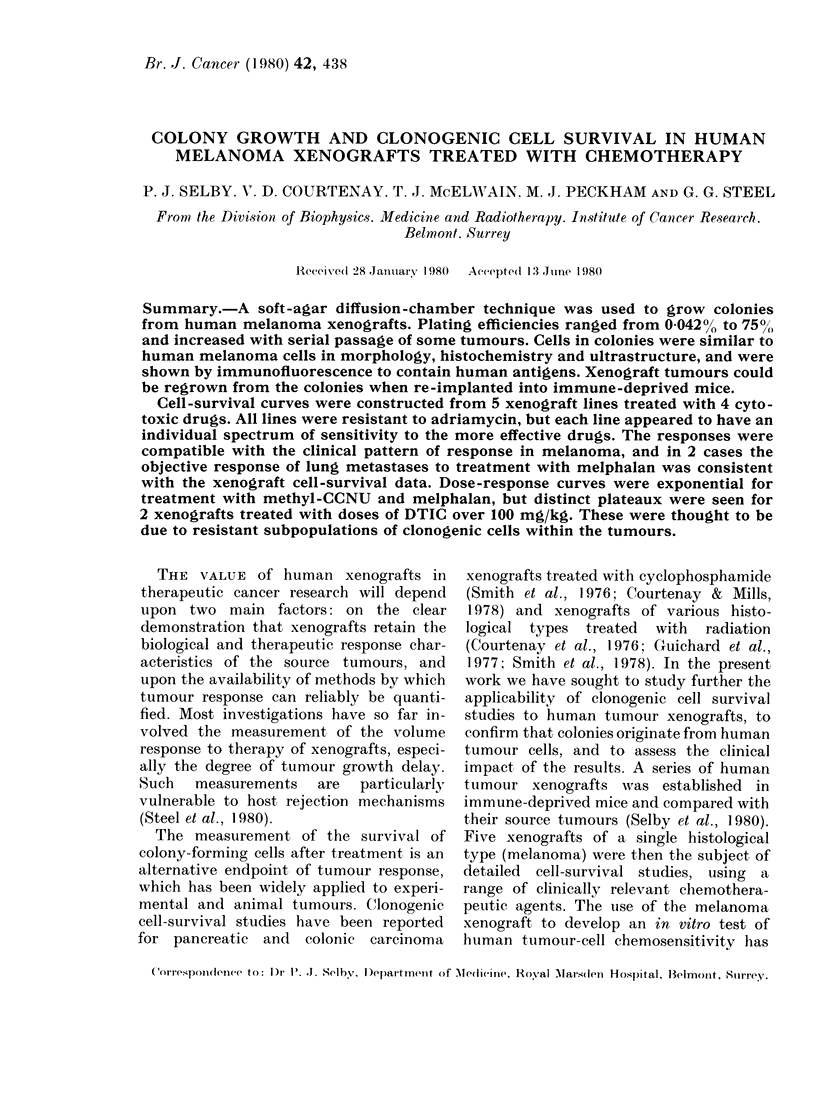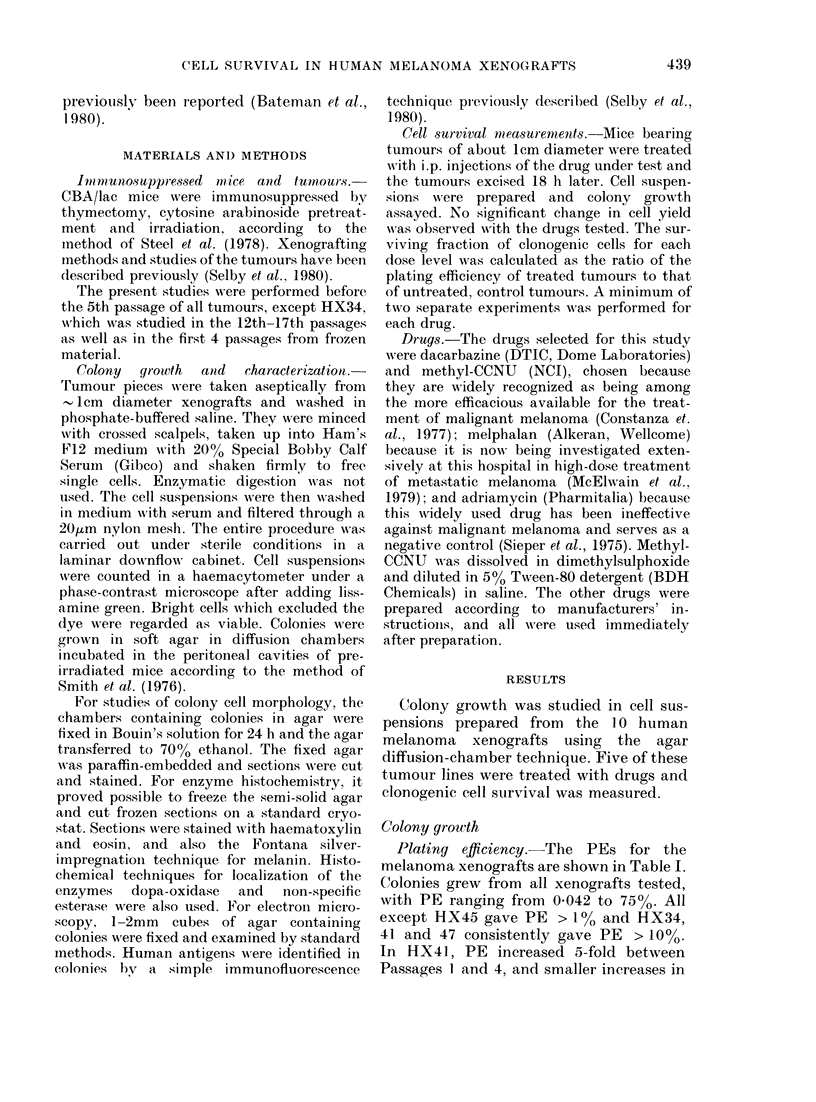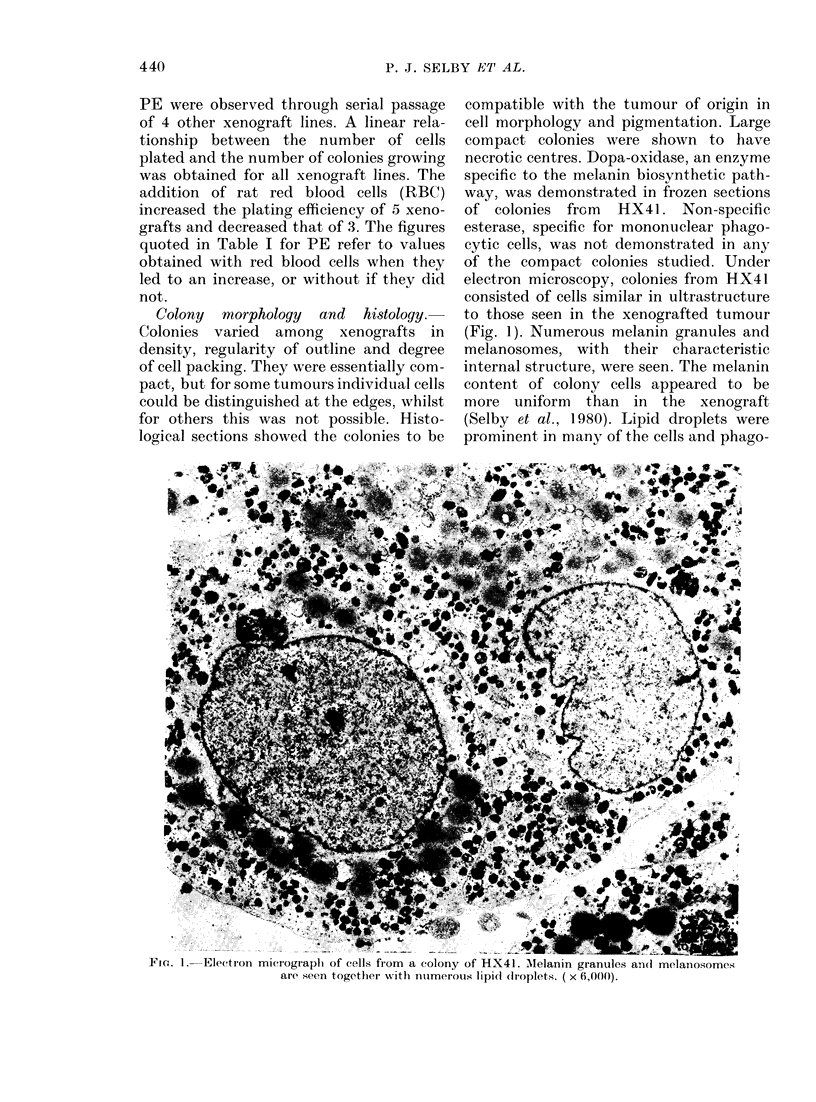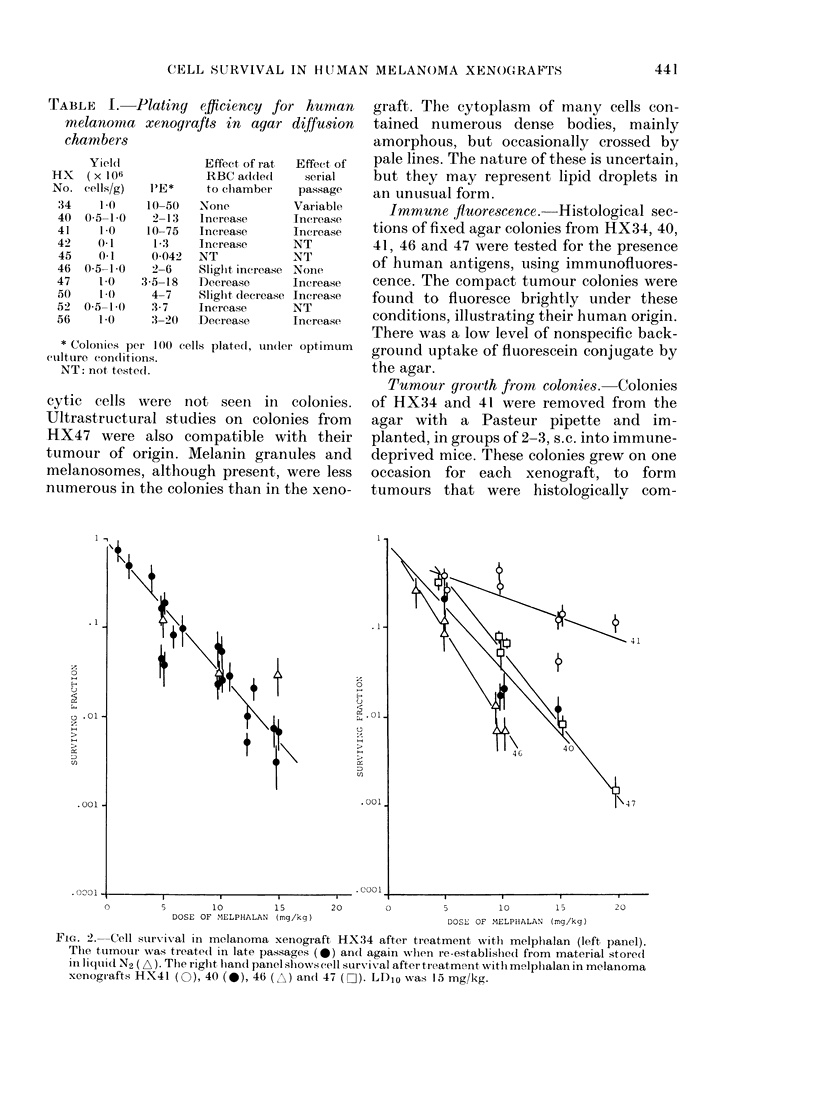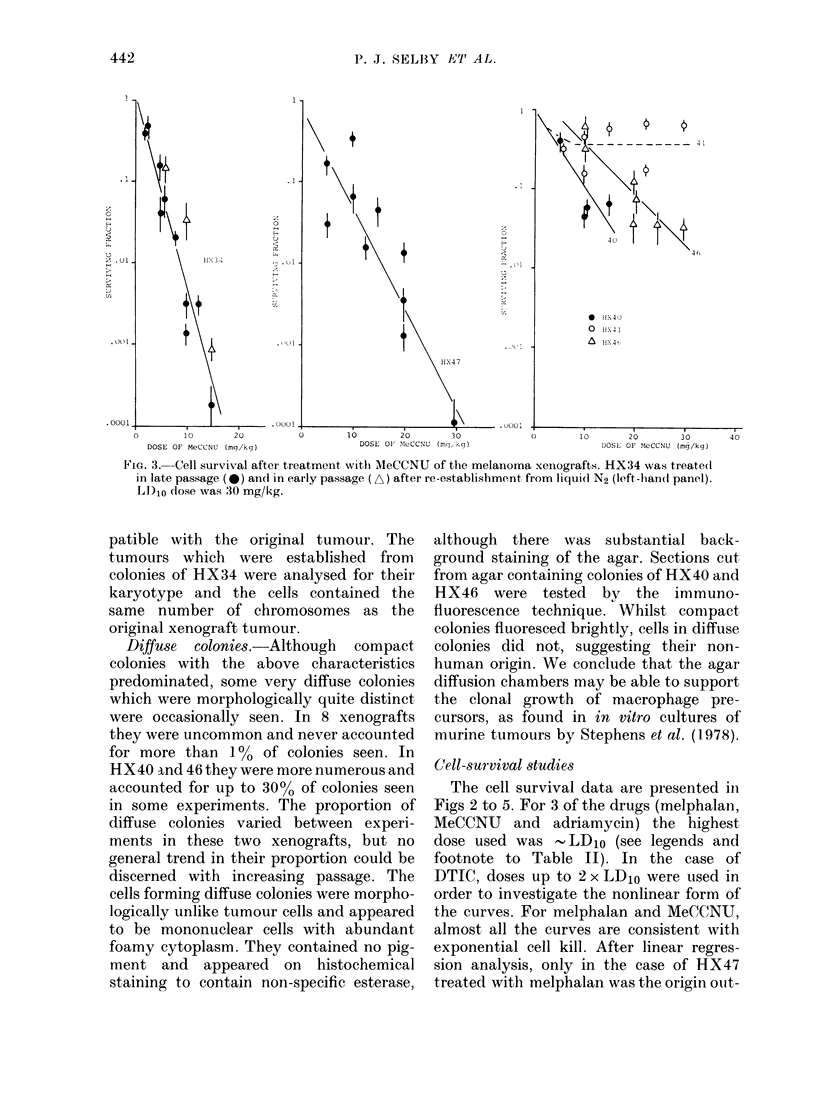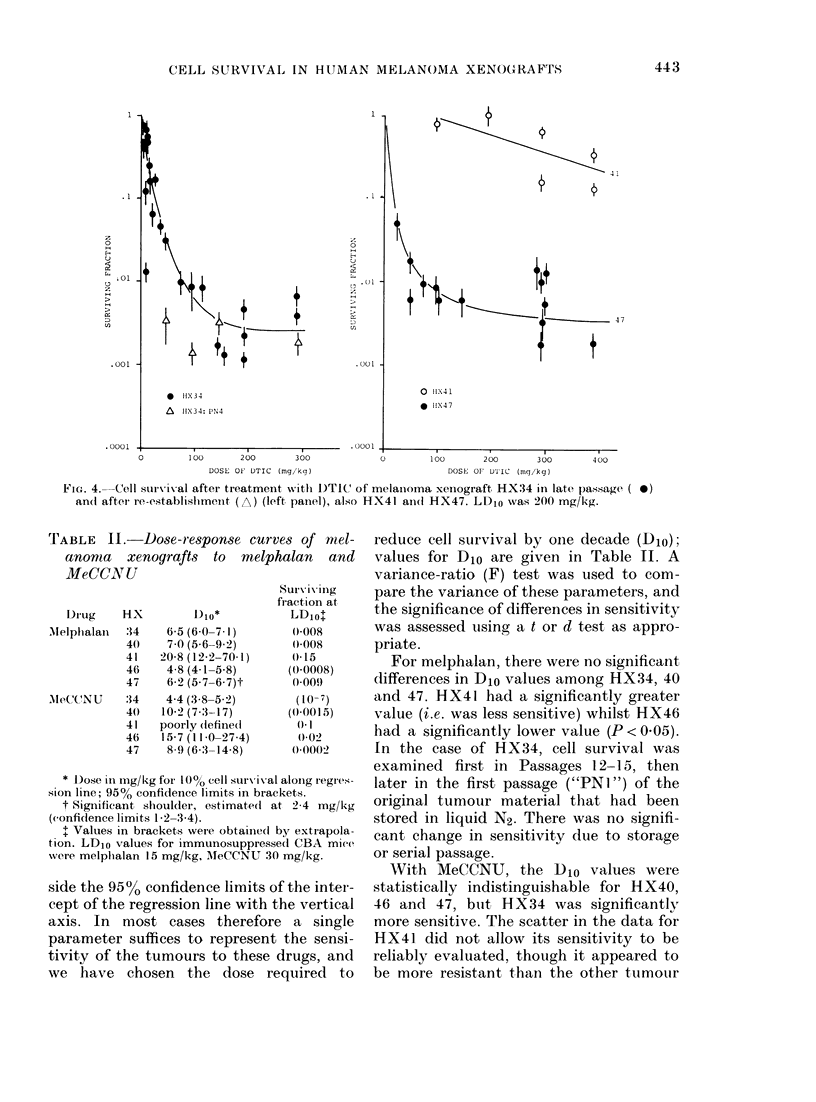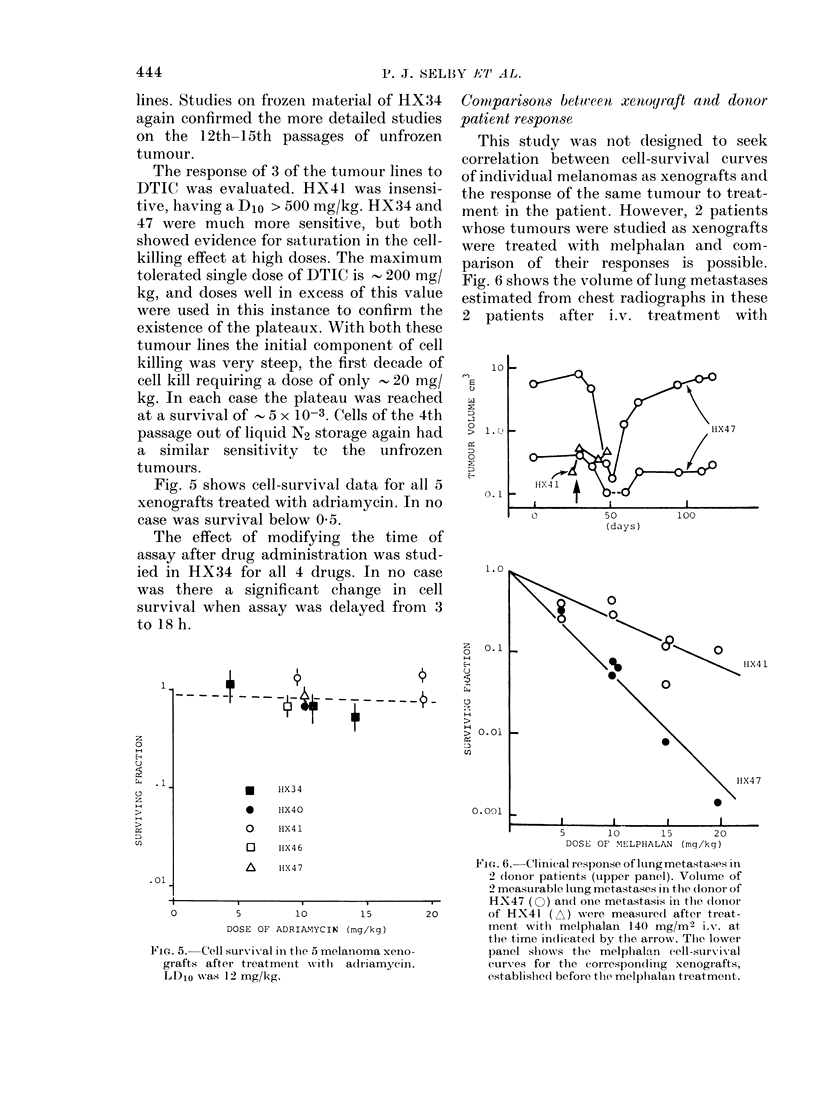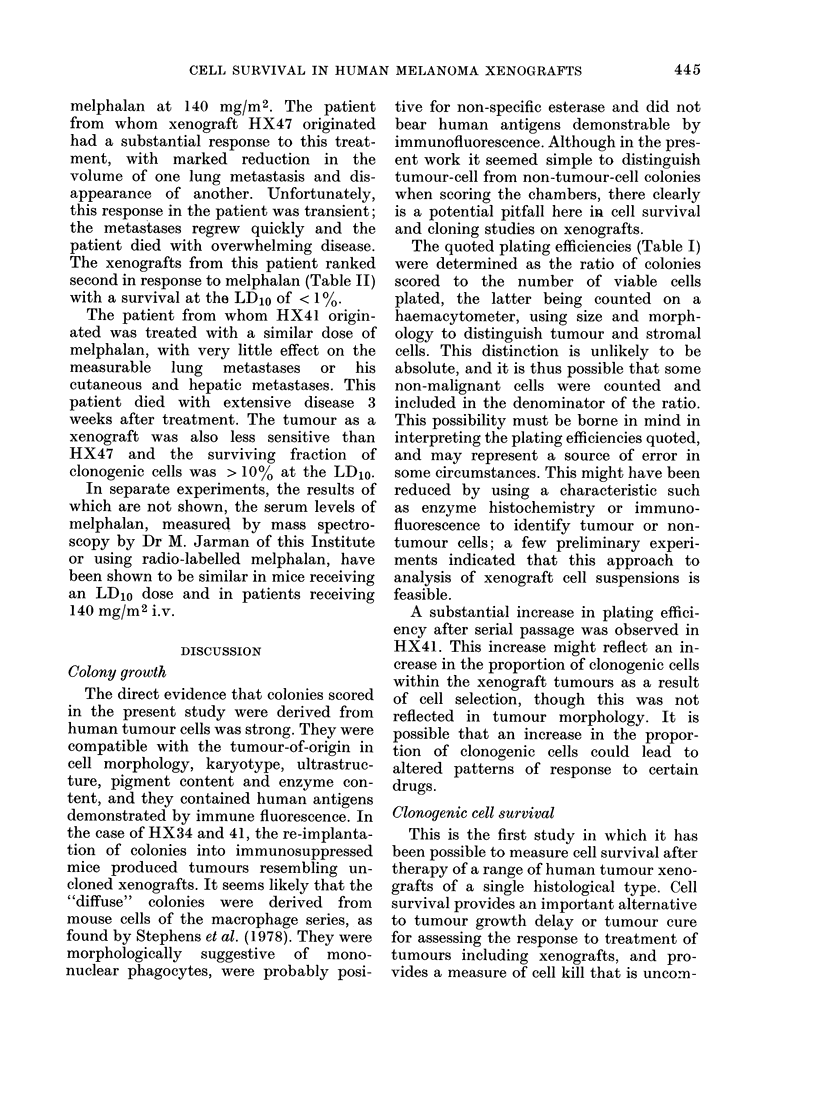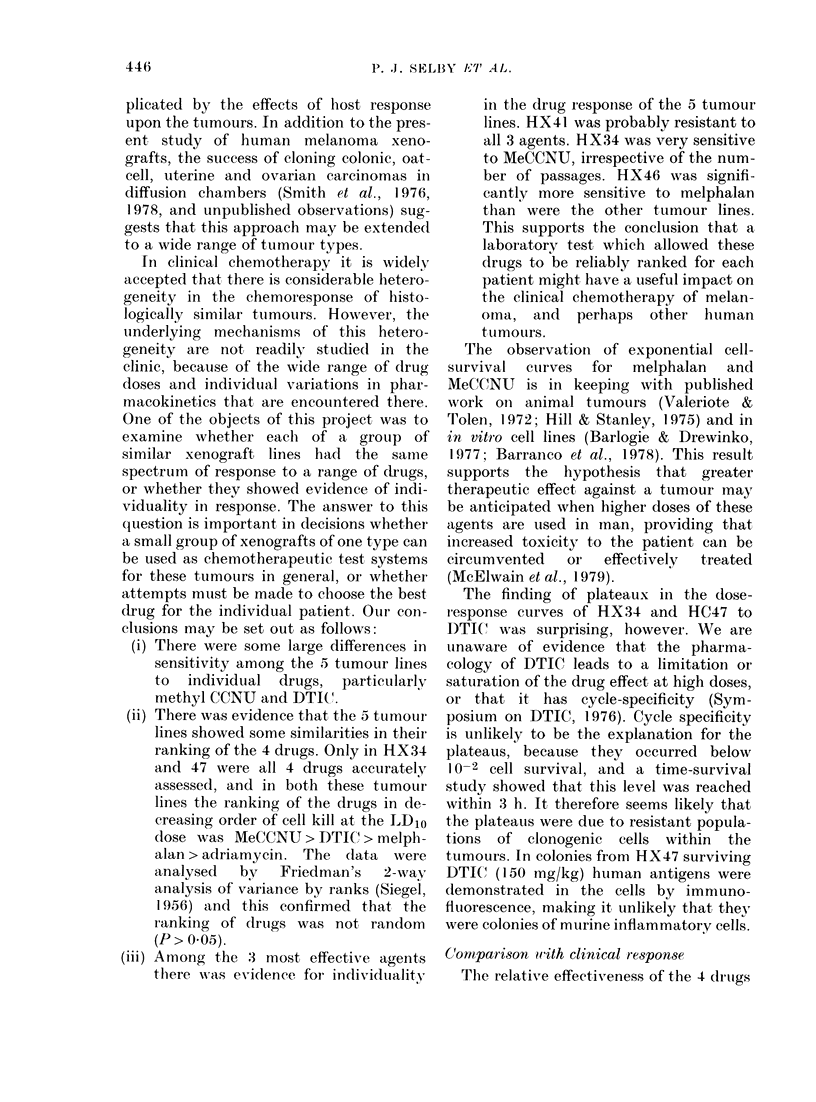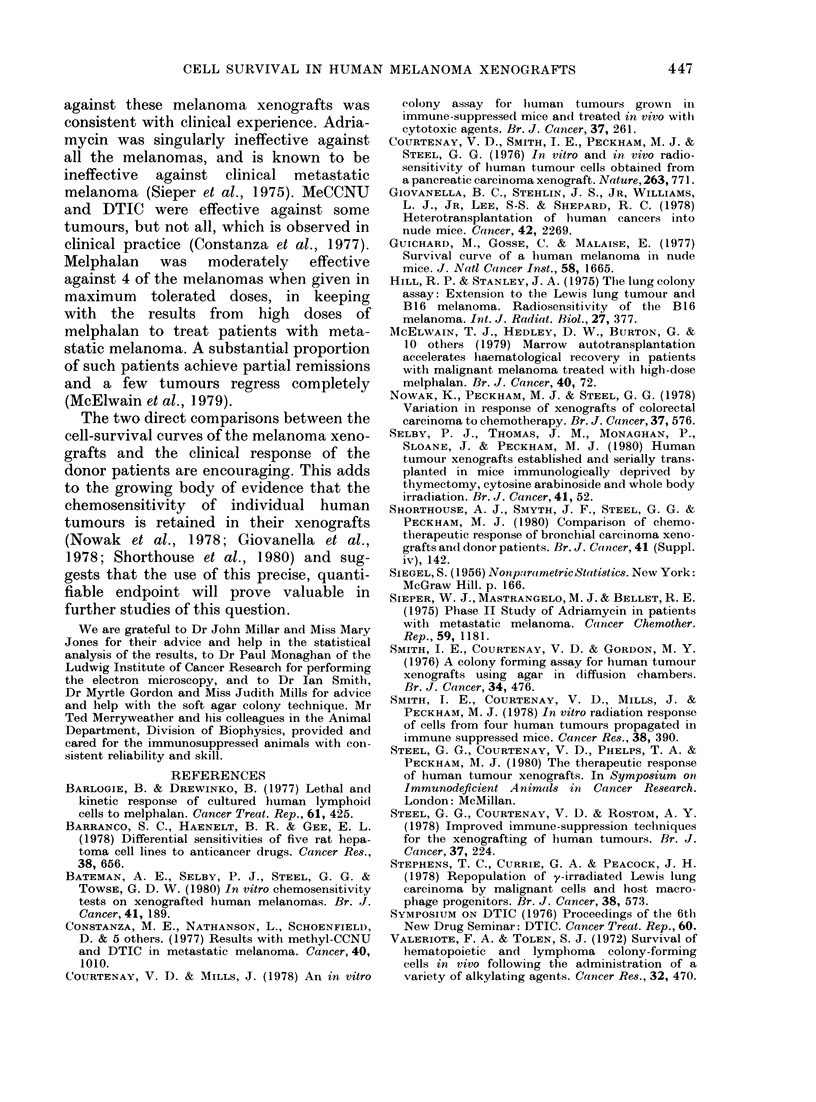# Colony growth and clonogenic cell survival in human melanoma xenografts treated with chemotherapy.

**DOI:** 10.1038/bjc.1980.256

**Published:** 1980-09

**Authors:** P. J. Selby, V. D. Courtenay, T. J. McElwain, M. J. Peckham, G. G. Steel

## Abstract

**Images:**


*Br. J. Cancer* (1980) **42**, 438

### COLONY GROWTH AND CLONOGENIC CELL SURVIVAL IN HUMAN MELANOMA XENOGRAFTS TREATED WITH CHEMOTHERAPY

P. J. SELBY, V. D. COURTENAY, T. J. McELWAIN, M. J. PECKHAM AND G. G. STEEL

*From the Division of Biophysics, Medicine and Radiotherapy, Institute of Cancer Research, Belmont, Surrey*

Received 28 January 1980  Accepted 13 June 1980

**Summary.—A soft-agar diffusion-chamber technique was used to grow colonies from human melanoma xenografts. Plating efficiencies ranged from 0·042% to 75% and increased with serial passage of some tumours. Cells in colonies were similar to human melanoma cells in morphology, histochemistry and ultrastructure, and were shown by immunofluorescence to contain human antigens. Xenograft tumours could be regrown from the colonies when re-implanted into immune-deprived mice.**

**Cell-survival curves were constructed from 5 xenograft lines treated with 4 cytotoxic drugs. All lines were resistant to adriamycin, but each line appeared to have an individual spectrum of sensitivity to the more effective drugs. The responses were compatible with the clinical pattern of response in melanoma, and in 2 cases the objective response of lung metastases to treatment with melphalan was consistent with the xenograft cell-survival data. Dose-response curves were exponential for treatment with methyl-CCNU and melphalan, but distinct plateaux were seen for 2 xenografts treated with doses of DTIC over 100 mg/kg. These were thought to be due to resistant subpopulations of clonogenic cells within the tumours.**

THE VALUE of human xenografts in therapeutic cancer research will depend upon two main factors: on the clear demonstration that xenografts retain the biological and therapeutic response characteristics of the source tumours, and upon the availability of methods by which tumour response can reliably be quantified. Most investigations have so far involved the measurement of the volume response to therapy of xenografts, especially the degree of tumour growth delay. Such measurements are particularly vulnerable to host rejection mechanisms (Steel *et al.*, 1980).

The measurement of the survival of colony-forming cells after treatment is an alternative endpoint of tumour response, which has been widely applied to experimental and animal tumours. Clonogenic cell-survival studies have been reported for pancreatic and colonic carcinoma xenografts treated with cyclophosphamide (Smith *et al.*, 1976; Courtenay & Mills, 1978) and xenografts of various histological types treated with radiation (Courtenay *et al.*, 1976; Guichard *et al.*, 1977; Smith *et al.*, 1978). In the present work we have sought to study further the applicability of clonogenic cell survival studies to human tumour xenografts, to confirm that colonies originate from human tumour cells, and to assess the clinical impact of the results. A series of human tumour xenografts was established in immune-deprived mice and compared with their source tumours (Selby *et al.*, 1980). Five xenografts of a single histological type (melanoma) were then the subject of detailed cell-survival studies, using a range of clinically relevant chemotherapeutic agents. The use of the melanoma xenograft to develop an *in vitro* test of human tumour-cell chemosensitivity has

Correspondence to: Dr P. J. Selby, Department of Medicine, Royal Marsden Hospital, Belmont, Surrey.

previously been reported (Bateman *et al.*, 1980).

MATERIALS AND METHODS

*Immunosuppressed mice and tumours.*—CBA/lac mice were immunosuppressed by thymectomy, cytosine arabinoside pretreatment and irradiation, according to the method of Steel *et al.* (1978). Xenografting methods and studies of the tumours have been described previously (Selby *et al.*, 1980).

The present studies were performed before the 5th passage of all tumours, except HX34, which was studied in the 12th–17th passages as well as in the first 4 passages from frozen material.

*Colony growth and characterization.*—Tumour pieces were taken aseptically from ~1cm diameter xenografts and washed in phosphate-buffered saline. They were minced with crossed scalpels, taken up into Ham's F12 medium with 20% Special Bobby Calf Serum (Gibco) and shaken firmly to free single cells. Enzymatic digestion was not used. The cell suspensions were then washed in medium with serum and filtered through a 20$\mu$m nylon mesh. The entire procedure was carried out under sterile conditions in a laminar downflow cabinet. Cell suspensions were counted in a haemacytometer under a phase-contrast microscope after adding lissamine green. Bright cells which excluded the dye were regarded as viable. Colonies were grown in soft agar in diffusion chambers incubated in the peritoneal cavities of pre-irradiated mice according to the method of Smith *et al.* (1976).

For studies of colony cell morphology, the chambers containing colonies in agar were fixed in Bouin's solution for 24 h and the agar transferred to 70% ethanol. The fixed agar was paraffin-embedded and sections were cut and stained. For enzyme histochemistry, it proved possible to freeze the semi-solid agar and cut frozen sections on a standard cryostat. Sections were stained with haematoxylin and eosin, and also the Fontana silver-impregnation technique for melanin. Histochemical techniques for localization of the enzymes dopa-oxidase and non-specific esterase were also used. For electron microscopy, 1–2mm cubes of agar containing colonies were fixed and examined by standard methods. Human antigens were identified in colonies by a simple immunofluorescence technique previously described (Selby *et al.*, 1980).

*Cell survival measurements.*—Mice bearing tumours of about 1cm diameter were treated with i.p. injections of the drug under test and the tumours excised 18 h later. Cell suspensions were prepared and colony growth assayed. No significant change in cell yield was observed with the drugs tested. The surviving fraction of clonogenic cells for each dose level was calculated as the ratio of the plating efficiency of treated tumours to that of untreated, control tumours. A minimum of two separate experiments was performed for each drug.

*Drugs.*—The drugs selected for this study were dacarbazine (DTIC, Dome Laboratories) and methyl-CCNU (NCI), chosen because they are widely recognized as being among the more efficacious available for the treatment of malignant melanoma (Constanza *et. al.*, 1977); melphalan (Alkeran, Wellcome) because it is now being investigated extensively at this hospital in high-dose treatment of metastatic melanoma (McElwain *et al.*, 1979); and adriamycin (Pharmitalia) because this widely used drug has been ineffective against malignant melanoma and serves as a negative control (Sieper *et al.*, 1975). Methyl-CCNU was dissolved in dimethylsulphoxide and diluted in 5% Tween-80 detergent (BDH Chemicals) in saline. The other drugs were prepared according to manufacturers' instructions, and all were used immediately after preparation.

RESULTS

Colony growth was studied in cell suspensions prepared from the 10 human melanoma xenografts using the agar diffusion-chamber technique. Five of these tumour lines were treated with drugs and clonogenic cell survival was measured.

*Colony growth*

*Plating efficiency.*—The PEs for the melanoma xenografts are shown in Table I. Colonies grew from all xenografts tested, with PE ranging from 0·042 to 75%. All except HX45 gave PE >1% and HX34, 41 and 47 consistently gave PE >10%. In HX41, PE increased 5-fold between Passages 1 and 4, and smaller increases in

PE were observed through serial passage of 4 other xenograft lines. A linear relationship between the number of cells plated and the number of colonies growing was obtained for all xenograft lines. The addition of rat red blood cells (RBC) increased the plating efficiency of 5 xenografts and decreased that of 3. The figures quoted in Table I for PE refer to values obtained with red blood cells when they led to an increase, or without if they did not.

*Colony morphology and histology.*—Colonies varied among xenografts in density, regularity of outline and degree of cell packing. They were essentially compact, but for some tumours individual cells could be distinguished at the edges, whilst for others this was not possible. Histological sections showed the colonies to be compatible with the tumour of origin in cell morphology and pigmentation. Large compact colonies were shown to have necrotic centres. Dopa-oxidase, an enzyme specific to the melanin biosynthetic pathway, was demonstrated in frozen sections of colonies from HX41. Non-specific esterase, specific for mononuclear phagocytic cells, was not demonstrated in any of the compact colonies studied. Under electron microscopy, colonies from HX41 consisted of cells similar in ultrastructure to those seen in the xenografted tumour (Fig. 1). Numerous melanin granules and melanosomes, with their characteristic internal structure, were seen. The melanin content of colony cells appeared to be more uniform than in the xenograft (Selby *et al.*, 1980). Lipid droplets were prominent in many of the cells and phago-

FIG. 1.—Electron micrograph of cells from a colony of HX41. Melanin granules and melanosomes are seen together with numerous lipid droplets. (× 6,000).

TABLE I.—*Plating efficiency for human melanoma xenografts in agar diffusion chambers*

| HX No. | Yield ($\times 10^6$ cells/g) | PE* | Effect of rat RBC added to chamber | Effect of serial passage |
|---|---|---|---|---|
| 34 | 1·0 | 10–50 | None | Variable |
| 40 | 0·5–1·0 | 2–13 | Increase | Increase |
| 41 | 1·0 | 10–75 | Increase | Increase |
| 42 | 0·1 | 1·3 | Increase | NT |
| 45 | 0·1 | 0·042 | NT | NT |
| 46 | 0·5–1·0 | 2–6 | Slight increase | None |
| 47 | 1·0 | 3·5–18 | Decrease | Increase |
| 50 | 1·0 | 4–7 | Slight decrease | Increase |
| 52 | 0·5–1·0 | 3·7 | Increase | NT |
| 56 | 1·0 | 3–20 | Decrease | Increase |

* Colonies per 100 cells plated, under optimum culture conditions.

NT: not tested.

cytic cells were not seen in colonies. Ultrastructural studies on colonies from HX47 were also compatible with their tumour of origin. Melanin granules and melanosomes, although present, were less numerous in the colonies than in the xenograft. The cytoplasm of many cells contained numerous dense bodies, mainly amorphous, but occasionally crossed by pale lines. The nature of these is uncertain, but they may represent lipid droplets in an unusual form.

*Immune fluorescence.*—Histological sections of fixed agar colonies from HX34, 40, 41, 46 and 47 were tested for the presence of human antigens, using immunofluorescence. The compact tumour colonies were found to fluoresce brightly under these conditions, illustrating their human origin. There was a low level of nonspecific background uptake of fluorescein conjugate by the agar.

*Tumour growth from colonies.*—Colonies of HX34 and 41 were removed from the agar with a Pasteur pipette and implanted, in groups of 2–3, s.c. into immune-deprived mice. These colonies grew on one occasion for each xenograft, to form tumours that were histologically com-

FIG. 2.—Cell survival in melanoma xenograft HX34 after treatment with melphalan (left panel). The tumour was treated in late passages (●) and again when re-established from material stored in liquid $N_2$ (△). The right hand panel shows cell survival after treatment with melphalan in melanoma xenografts HX41 (○), 40 (●), 46 (△) and 47 (□). $LD_{10}$ was 15 mg/kg.

FIG. 3.—Cell survival after treatment with MeCCNU of the melanoma xenografts. HX34 was treated in late passage (●) and in early passage (△) after re-establishment from liquid $N_2$ (left-hand panel). $LD_{10}$ dose was 30 mg/kg.

patible with the original tumour. The tumours which were established from colonies of HX34 were analysed for their karyotype and the cells contained the same number of chromosomes as the original xenograft tumour.

*Diffuse colonies.*—Although compact colonies with the above characteristics predominated, some very diffuse colonies which were morphologically quite distinct were occasionally seen. In 8 xenografts they were uncommon and never accounted for more than 1% of colonies seen. In HX40 and 46 they were more numerous and accounted for up to 30% of colonies seen in some experiments. The proportion of diffuse colonies varied between experiments in these two xenografts, but no general trend in their proportion could be discerned with increasing passage. The cells forming diffuse colonies were morphologically unlike tumour cells and appeared to be mononuclear cells with abundant foamy cytoplasm. They contained no pigment and appeared on histochemical staining to contain non-specific esterase, although there was substantial background staining of the agar. Sections cut from agar containing colonies of HX40 and HX46 were tested by the immunofluorescence technique. Whilst compact colonies fluoresced brightly, cells in diffuse colonies did not, suggesting their non-human origin. We conclude that the agar diffusion chambers may be able to support the clonal growth of macrophage precursors, as found in *in vitro* cultures of murine tumours by Stephens *et al.* (1978).

*Cell-survival studies*

The cell survival data are presented in Figs 2 to 5. For 3 of the drugs (melphalan, MeCCNU and adriamycin) the highest dose used was $\sim LD_{10}$ (see legends and footnote to Table II). In the case of DTIC, doses up to $2 \times LD_{10}$ were used in order to investigate the nonlinear form of the curves. For melphalan and MeCCNU, almost all the curves are consistent with exponential cell kill. After linear regression analysis, only in the case of HX47 treated with melphalan was the origin out-

FIG. 4.—Cell survival after treatment with DTIC of melanoma xenograft HX34 in late passage (●) and after re-establishment (△) (left panel), also HX41 and HX47. $LD_{10}$ was 200 mg/kg.

TABLE II.—*Dose-response curves of melanoma xenografts to melphalan and MeCCNU*

| Drug | HX | $D_{10}$* | Surviving fraction at $LD_{10}$‡ |
|---|---|---|---|
| Melphalan | 34 | 6·5 (6·0–7·1) | 0·008 |
| | 40 | 7·0 (5·6–9·2) | 0·008 |
| | 41 | 20·8 (12·2–70·1) | 0·15 |
| | 46 | 4·8 (4·1–5·8) | (0·0008) |
| | 47 | 6·2 (5·7–6·7)† | 0·009 |
| MeCCNU | 34 | 4·4 (3·8–5·2) | ($10^{-7}$) |
| | 40 | 10·2 (7·3–17) | (0·0015) |
| | 41 | poorly defined | 0·1 |
| | 46 | 15·7 (11·0–27·4) | 0·02 |
| | 47 | 8·9 (6·3–14·8) | 0·0002 |

* Dose in mg/kg for 10% cell survival along regression line; 95% confidence limits in brackets.

† Significant shoulder, estimated at 2·4 mg/kg (confidence limits 1·2–3·4).

‡ Values in brackets were obtained by extrapolation. $LD_{10}$ values for immunosuppressed CBA mice were melphalan 15 mg/kg, MeCCNU 30 mg/kg.

side the 95% confidence limits of the intercept of the regression line with the vertical axis. In most cases therefore a single parameter suffices to represent the sensitivity of the tumours to these drugs, and we have chosen the dose required to reduce cell survival by one decade ($D_{10}$); values for $D_{10}$ are given in Table II. A variance-ratio (F) test was used to compare the variance of these parameters, and the significance of differences in sensitivity was assessed using a *t* or *d* test as appropriate.

For melphalan, there were no significant differences in $D_{10}$ values among HX34, 40 and 47. HX41 had a significantly greater value (*i.e.* was less sensitive) whilst HX46 had a significantly lower value ($P < 0·05$). In the case of HX34, cell survival was examined first in Passages 12–15, then later in the first passage ("PN1") of the original tumour material that had been stored in liquid $N_2$. There was no significant change in sensitivity due to storage or serial passage.

With MeCCNU, the $D_{10}$ values were statistically indistinguishable for HX40, 46 and 47, but HX34 was significantly more sensitive. The scatter in the data for HX41 did not allow its sensitivity to be reliably evaluated, though it appeared to be more resistant than the other tumour lines. Studies on frozen material of HX34 again confirmed the more detailed studies on the 12th–15th passages of unfrozen tumour.

The response of 3 of the tumour lines to DTIC was evaluated. HX41 was insensitive, having a $D_{10} > 500$ mg/kg. HX34 and 47 were much more sensitive, but both showed evidence for saturation in the cell-killing effect at high doses. The maximum tolerated single dose of DTIC is $\sim 200$ mg/kg, and doses well in excess of this value were used in this instance to confirm the existence of the plateaux. With both these tumour lines the initial component of cell killing was very steep, the first decade of cell kill requiring a dose of only $\sim 20$ mg/kg. In each case the plateau was reached at a survival of $\sim 5 \times 10^{-3}$. Cells of the 4th passage out of liquid $N_2$ storage again had a similar sensitivity to the unfrozen tumours.

Fig. 5 shows cell-survival data for all 5 xenografts treated with adriamycin. In no case was survival below 0·5.

The effect of modifying the time of assay after drug administration was studied in HX34 for all 4 drugs. In no case was there a significant change in cell survival when assay was delayed from 3 to 18 h.

FIG. 5.—Cell survival in the 5 melanoma xenografts after treatment with adriamycin. $LD_{10}$ was 12 mg/kg.

*Comparisons between xenograft and donor patient response*

This study was not designed to seek correlation between cell-survival curves of individual melanomas as xenografts and the response of the same tumour to treatment in the patient. However, 2 patients whose tumours were studied as xenografts were treated with melphalan and comparison of their responses is possible. Fig. 6 shows the volume of lung metastases estimated from chest radiographs in these 2 patients after i.v. treatment with

FIG. 6.—Clinical response of lung metastases in 2 donor patients (upper panel). Volume of 2 measurable lung metastases in the donor of HX47 (○) and one metastasis in the donor of HX41 (△) were measured after treatment with melphalan 140 mg/m² i.v. at the time indicated by the arrow. The lower panel shows the melphalan cell-survival curves for the corresponding xenografts, established before the melphalan treatment.

melphalan at 140 mg/m$^2$. The patient from whom xenograft HX47 originated had a substantial response to this treatment, with marked reduction in the volume of one lung metastasis and disappearance of another. Unfortunately, this response in the patient was transient; the metastases regrew quickly and the patient died with overwhelming disease. The xenografts from this patient ranked second in response to melphalan (Table II) with a survival at the $LD_{10}$ of $< 1\%$.

The patient from whom HX41 originated was treated with a similar dose of melphalan, with very little effect on the measurable lung metastases or his cutaneous and hepatic metastases. This patient died with extensive disease 3 weeks after treatment. The tumour as a xenograft was also less sensitive than HX47 and the surviving fraction of clonogenic cells was $> 10\%$ at the $LD_{10}$.

In separate experiments, the results of which are not shown, the serum levels of melphalan, measured by mass spectroscopy by Dr M. Jarman of this Institute or using radio-labelled melphalan, have been shown to be similar in mice receiving an $LD_{10}$ dose and in patients receiving 140 mg/m$^2$ i.v.

### DISCUSSION

### *Colony growth*

The direct evidence that colonies scored in the present study were derived from human tumour cells was strong. They were compatible with the tumour-of-origin in cell morphology, karyotype, ultrastructure, pigment content and enzyme content, and they contained human antigens demonstrated by immune fluorescence. In the case of HX34 and 41, the re-implantation of colonies into immunosuppressed mice produced tumours resembling uncloned xenografts. It seems likely that the "diffuse" colonies were derived from mouse cells of the macrophage series, as found by Stephens *et al.* (1978). They were morphologically suggestive of mononuclear phagocytes, were probably positive for non-specific esterase and did not bear human antigens demonstrable by immunofluorescence. Although in the present work it seemed simple to distinguish tumour-cell from non-tumour-cell colonies when scoring the chambers, there clearly is a potential pitfall here in cell survival and cloning studies on xenografts.

The quoted plating efficiencies (Table I) were determined as the ratio of colonies scored to the number of viable cells plated, the latter being counted on a haemacytometer, using size and morphology to distinguish tumour and stromal cells. This distinction is unlikely to be absolute, and it is thus possible that some non-malignant cells were counted and included in the denominator of the ratio. This possibility must be borne in mind in interpreting the plating efficiencies quoted, and may represent a source of error in some circumstances. This might have been reduced by using a characteristic such as enzyme histochemistry or immunofluorescence to identify tumour or non-tumour cells; a few preliminary experiments indicated that this approach to analysis of xenograft cell suspensions is feasible.

A substantial increase in plating efficiency after serial passage was observed in HX41. This increase might reflect an increase in the proportion of clonogenic cells within the xenograft tumours as a result of cell selection, though this was not reflected in tumour morphology. It is possible that an increase in the proportion of clonogenic cells could lead to altered patterns of response to certain drugs.

### *Clonogenic cell survival*

This is the first study in which it has been possible to measure cell survival after therapy of a range of human tumour xenografts of a single histological type. Cell survival provides an important alternative to tumour growth delay or tumour cure for assessing the response to treatment of tumours including xenografts, and provides a measure of cell kill that is uncomplicated by the effects of host response upon the tumours. In addition to the present study of human melanoma xenografts, the success of cloning colonic, oatcell, uterine and ovarian carcinomas in diffusion chambers (Smith *et al.*, 1976, 1978, and unpublished observations) suggests that this approach may be extended to a wide range of tumour types.

In clinical chemotherapy it is widely accepted that there is considerable heterogeneity in the chemoresponse of histologically similar tumours. However, the underlying mechanisms of this heterogeneity are not readily studied in the clinic, because of the wide range of drug doses and individual variations in pharmacokinetics that are encountered there. One of the objects of this project was to examine whether each of a group of similar xenograft lines had the same spectrum of response to a range of drugs, or whether they showed evidence of individuality in response. The answer to this question is important in decisions whether a small group of xenografts of one type can be used as chemotherapeutic test systems for these tumours in general, or whether attempts must be made to choose the best drug for the individual patient. Our conclusions may be set out as follows:

(i) There were some large differences in sensitivity among the 5 tumour lines to individual drugs, particularly methyl CCNU and DTIC.

(ii) There was evidence that the 5 tumour lines showed some similarities in their ranking of the 4 drugs. Only in HX34 and 47 were all 4 drugs accurately assessed, and in both these tumour lines the ranking of the drugs in decreasing order of cell kill at the $LD_{10}$ dose was MeCCNU > DTIC > melphalan > adriamycin. The data were analysed by Friedman's 2-way analysis of variance by ranks (Siegel, 1956) and this confirmed that the ranking of drugs was not random ($P > 0{\cdot}05$).

(iii) Among the 3 most effective agents there was evidence for individuality in the drug response of the 5 tumour lines. HX41 was probably resistant to all 3 agents. HX34 was very sensitive to MeCCNU, irrespective of the number of passages. HX46 was significantly more sensitive to melphalan than were the other tumour lines. This supports the conclusion that a laboratory test which allowed these drugs to be reliably ranked for each patient might have a useful impact on the clinical chemotherapy of melanoma, and perhaps other human tumours.

The observation of exponential cell-survival curves for melphalan and MeCCNU is in keeping with published work on animal tumours (Valeriote & Tolen, 1972; Hill & Stanley, 1975) and in *in vitro* cell lines (Barlogie & Drewinko, 1977; Barranco *et al.*, 1978). This result supports the hypothesis that greater therapeutic effect against a tumour may be anticipated when higher doses of these agents are used in man, providing that increased toxicity to the patient can be circumvented or effectively treated (McElwain *et al.*, 1979).

The finding of plateaux in the dose-response curves of HX34 and HC47 to DTIC was surprising, however. We are unaware of evidence that the pharmacology of DTIC leads to a limitation or saturation of the drug effect at high doses, or that it has cycle-specificity (Symposium on DTIC, 1976). Cycle specificity is unlikely to be the explanation for the plateaus, because they occurred below $10^{-2}$ cell survival, and a time-survival study showed that this level was reached within 3 h. It therefore seems likely that the plateaus were due to resistant populations of clonogenic cells within the tumours. In colonies from HX47 surviving DTIC (150 mg/kg) human antigens were demonstrated in the cells by immunofluorescence, making it unlikely that they were colonies of murine inflammatory cells.

*Comparison with clinical response*

The relative effectiveness of the 4 drugs against these melanoma xenografts was consistent with clinical experience. Adriamycin was singularly ineffective against all the melanomas, and is known to be ineffective against clinical metastatic melanoma (Sieper *et al.*, 1975). MeCCNU and DTIC were effective against some tumours, but not all, which is observed in clinical practice (Constanza *et al.*, 1977). Melphalan was moderately effective against 4 of the melanomas when given in maximum tolerated doses, in keeping with the results from high doses of melphalan to treat patients with metastatic melanoma. A substantial proportion of such patients achieve partial remissions and a few tumours regress completely (McElwain *et al.*, 1979).

The two direct comparisons between the cell-survival curves of the melanoma xenografts and the clinical response of the donor patients are encouraging. This adds to the growing body of evidence that the chemosensitivity of individual human tumours is retained in their xenografts (Nowak *et al.*, 1978; Giovanella *et al.*, 1978; Shorthouse *et al.*, 1980) and suggests that the use of this precise, quantifiable endpoint will prove valuable in further studies of this question.

We are grateful to Dr John Millar and Miss Mary Jones for their advice and help in the statistical analysis of the results, to Dr Paul Monaghan of the Ludwig Institute of Cancer Research for performing the electron microscopy, and to Dr Ian Smith, Dr Myrtle Gordon and Miss Judith Mills for advice and help with the soft agar colony technique. Mr Ted Merryweather and his colleagues in the Animal Department, Division of Biophysics, provided and cared for the immunosuppressed animals with consistent reliability and skill.

### REFERENCES

BARLOGIE, B. & DREWINKO, B. (1977) Lethal and kinetic response of cultured human lymphoid cells to melphalan. *Cancer Treat. Rep.*, **61**, 425.

BARRANCO, S. C., HAENELT, B. R. & GEE, E. L. (1978) Differential sensitivities of five rat hepatoma cell lines to anticancer drugs. *Cancer Res.*, **38**, 656.

BATEMAN, A. E., SELBY, P. J., STEEL, G. G. & TOWSE, G. D. W. (1980) *In vitro* chemosensitivity tests on xenografted human melanomas. *Br. J. Cancer*, **41**, 189.

CONSTANZA, M. E., NATHANSON, L., SCHOENFIELD, D. & 5 others. (1977) Results with methyl-CCNU and DTIC in metastatic melanoma. *Cancer*, **40**, 1010.

COURTENAY, V. D. & MILLS, J. (1978) An *in vitro* colony assay for human tumours grown in immune-suppressed mice and treated *in vivo* with cytotoxic agents. *Br. J. Cancer*, **37**, 261.

COURTENAY, V. D., SMITH, I. E., PECKHAM, M. J. & STEEL, G. G. (1976) *In vitro* and *in vivo* radiosensitivity of human tumour cells obtained from a pancreatic carcinoma xenograft. *Nature*, **263**, 771.

GIOVANELLA, B. C., STEHLIN, J. S., JR, WILLIAMS, L. J., JR, LEE, S-S. & SHEPARD, R. C. (1978) Heterotransplantation of human cancers into nude mice. *Cancer*, **42**, 2269.

GUICHARD, M., GOSSE, C. & MALAISE, E. (1977) Survival curve of a human melanoma in nude mice. *J. Natl Cancer Inst.*, **58**, 1665.

HILL, R. P. & STANLEY, J. A. (1975) The lung colony assay: Extension to the Lewis lung tumour and B16 melanoma. Radiosensitivity of the B16 melanoma. *Int. J. Radiat. Biol.*, **27**, 377.

MCELWAIN, T. J., HEDLEY, D. W., BURTON, G. & 10 others (1979) Marrow autotransplantation accelerates haematological recovery in patients with malignant melanoma treated with high-dose melphalan. *Br. J. Cancer*, **40**, 72.

NOWAK, K., PECKHAM, M. J. & STEEL, G. G. (1978) Variation in response of xenografts of colorectal carcinoma to chemotherapy. *Br. J. Cancer*, **37**, 576.

SELBY, P. J., THOMAS, J. M., MONAGHAN, P., SLOANE, J. & PECKHAM, M. J. (1980) Human tumour xenografts established and serially transplanted in mice immunologically deprived by thymectomy, cytosine arabinoside and whole body irradiation. *Br. J. Cancer*, **41**, 52.

SHORTHOUSE, A. J., SMYTH, J. F., STEEL, G. G. & PECKHAM, M. J. (1980) Comparison of chemotherapeutic response of bronchial carcinoma xenografts and donor patients. *Br. J. Cancer*, **41** (Suppl. iv), 142.

SIEGEL, S. (1956) *Nonparametric Statistics.* New York: McGraw Hill. p. 166.

SIEPER, W. J., MASTRANGELO, M. J. & BELLET, R. E. (1975) Phase II Study of Adriamycin in patients with metastatic melanoma. *Cancer Chemother. Rep.*, **59**, 1181.

SMITH, I. E., COURTENAY, V. D. & GORDON, M. Y. (1976) A colony forming assay for human tumour xenografts using agar in diffusion chambers. *Br. J. Cancer*, **34**, 476.

SMITH, I. E., COURTENAY, V. D., MILLS, J. & PECKHAM, M. J. (1978) *In vitro* radiation response of cells from four human tumours propagated in immune suppressed mice. *Cancer Res.*, **38**, 390.

STEEL, G. G., COURTENAY, V. D., PHELPS, T. A. & PECKHAM, M. J. (1980) The therapeutic response of human tumour xenografts. In *Symposium on Immunodeficient Animals in Cancer Research.* London: McMillan.

STEEL, G. G., COURTENAY, V. D. & ROSTOM, A. Y. (1978) Improved immune-suppression techniques for the xenografting of human tumours. *Br. J. Cancer*, **37**, 224.

STEPHENS, T. C., CURRIE, G. A. & PEACOCK, J. H. (1978) Repopulation of $\gamma$-irradiated Lewis lung carcinoma by malignant cells and host macrophage progenitors. *Br. J. Cancer*, **38**, 573.

SYMPOSIUM ON DTIC (1976) Proceedings of the 6th New Drug Seminar: DTIC. *Cancer Treat. Rep.*, **60**.

VALERIOTE, F. A. & TOLEN, S. J. (1972) Survival of hematopoietic and lymphoma colony-forming cells *in vivo* following the administration of a variety of alkylating agents. *Cancer Res.*, **32**, 470.